# Risk Factors of Transient Cortical Blindness After Cerebral Angiography: A Multicenter Study

**DOI:** 10.3389/fneur.2019.01005

**Published:** 2019-09-18

**Authors:** Miao Li, Huaxin Liang, Chao Liu, Hongtao Liu, Yang Zheng, Wanchao Shi, Jie Wang

**Affiliations:** ^1^Department of Neurosurgery, The China-Japan Union Hospital of Jilin University, Changchun, China; ^2^Department of Neurosurgery, The Second Hospital of Jilin University, Changchun, China; ^3^Department of Neurosurgery, Jilin Central Hospital, Jilin, China; ^4^Department of Neurosurgery, Siping Central Hospital, Siping, China; ^5^Department of Neurosurgery, Tianjin Fifth Central Hospital, Tianjin, China; ^6^Department of Neurology, The China-Japan Union Hospital of Jilin University, Changchun, China

**Keywords:** transient cortical blindness, cerebral angiography, contrast agent dose, weight, digital subtraction angiography

## Abstract

**Background:** Although transient cortical blindness is a rare complication following cerebral angiography, identification of risk factors for the development of transient cortical blindness after cerebral angiography is an important clinical issue.

**Material and methods:** Between January 2008 and April 2018, 5,126 patients at five high-volume medical centers who underwent cerebral angiography procedures were enrolled in this multicenter cohort study. Patient baseline characteristics and surgery-related factors were analyzed. We used multivariate logistic regression to examine factors associated with transient cortical blindness.

**Results:** Eighteen patients (0.35%) in the total cohort of 5,126 suffered transient cortical blindness. After univariate statistical analysis, no significant differences were determined between the transient cortical blindness group and the control group regarding gender (*p* = 0.454), age (*p* = 0.872), smoking (*p* = 0.170), diabetes (*p* = 0.800), and hypertension (*p* = 0.100). Compared with the control group, the transient cortical blindness group weighed less (*p* = 0.020), and had a larger dose of contrast agent (*p* = 0.034) and more instances of contrast agent injected into the posterior circulation (*p* < 0.001). Logistic regression analysis identified contrast agent dose and contrast agent injected into posterior circulation as independent predictive factors for transient cortical blindness (*P* < 0.05).

**Conclusion:** Larger doses off contrast agent and contrast agent injected into the posterior circulation are potential independent predictive factors for transient cortical blindness following cerebral angiography.

## Introduction

Cerebral angiography is frequently performed, with digital subtraction angiography (DSA) being the gold standard for the detection of vascular diseases of the brain. Transient cortical blindness, a rare complication following administration of a contrast agent, has a reported incidence of 0.3–1% after cerebral angiography (1, 2). Identifying the risk factors for transient cortical blindness after cerebral angiography remains an important issue and would provide helpful reference points for physicians. However, most authors of previous studies of transient cortical blindness have drawn their conclusion on the basis of single-center data and a limited number of cases (3–5). This retrospective study employed a large multicenter sample of patients to identify the risk factors related to transient cortical blindness after cerebral angiography. We examined baseline information and the details of cerebral angiography in 5,126 consecutive patients at five high-volume medical centers. To the best of our knowledge, this is the largest sample size used thus far to identify such risk factors.

## Materials and Methods

### Selection of Patients and Population

**Figure 1:**
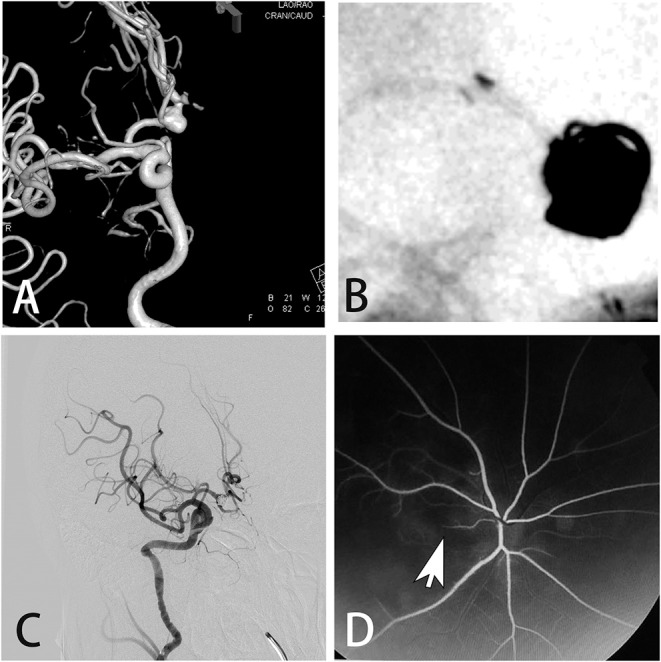
A 51-year-old male presented with an asymptomatic aneurysm. **(A)** Digital subtraction angiography showed the aneurysm on the anterior communicating artery. **(B)** The aneurysm was treated by stent assisted coiling. **(C)** Immediate angiogram after the procedure showed the complete occlusion of the aneurysm. However, the patient suffered blindness of the right eye. **(D)** Fluorescence fundus angiography showed the occlusion of the branch of the fundus oculi artery.

This retrospective study was approved by the Institutional Review Board and was carried out in five research units. From January 2008 to April 2018, a total of 5,126 patients who underwent cerebral angiography were enrolled according to the following criteria: (1) patients who were diagnosed with cervical and intracranial vascular diseases; (2) patients aged 18–75 years; and (3) patients who underwent cerebral angiography. The exclusion criteria were: (1) patients with severely abnormal lung, kidney, or liver function; (2) patients with hyperthyroidism; (3) patients with coagulation disorders; and (4) patients with mental illness. The following factors were analyzed: age, sex, weight, cigarette smoking, hypertension, diabetes, dose of contrast agent, and delivery of contrast agent to posterior circulation. Patients were diagnosed with transient cortical blindness according to the following criteria: (1) simultaneous loss of vision in both eyes with sudden onset, which could be completely restored and was not associated with an intervention and definite symptoms of focal cerebral ischemia, epilepsy, migraine, or change of consciousness; (2) the eyes themselves (i.e., media, retina, and optic nerve) were normal or showed only minor abnormality, incapable of causing blindness; and (3) the pupillary light reactions were normal (6, 7). Neurological and ophthalmological investigations (such as CT, MRI, electroretinogram, and visual evoked potential) were performed for patients who suffered blindness after cerebral angiography. The patient shown in Figure 1 suffered blindness of the right eye caused by occlusion of the branch of the fundus oculi artery and so could not be diagnosed as having transient cortical blindness.

### Cerebral Angiography Procedures

All procedures were performed using the Seldinger technique with a 5F or 6F artery sheath, preceded by local anesthesia with 1% lignocaine solution. A nonionic low-osmolar contrast material (ioversol, 320 mg/mL) was used for the angiography. The dose and rate of contrast injection were conducted in accordance with the following criteria. For the vertebrobasilar arterial system, the contrast was administered at the rate of 3 mL/s, with 5 mL in total for 2-dimensional (2D) DSA or 18 mL in total for 3-dimensional (3D) DSA. For the internal carotid arterial system, the contrast was administered at the rate of 4 mL/s, with 6 mL in total for 2D DSA or 24 mL in total for 3D DSA. During the procedure, systemic heparinization was applied with a loading dose of 3,000 IU of heparin with 500 mL of normal saline. At the end of the procedure, the artery sheath was removed. Hemostasis by compression was performed by a femoral artery compression device or elastic bandage.

### Treatment of Transient Cortical Blindness

For the treatment of patients with cortical blindness in this study, the main principles were dilation of cerebral vessels, alleviation of cerebral edema, volume expansion, and steroids. Therefore, nimodipine (10 mg) (Bayer Pharma, Germany; J20140105) was continuously given intravenously at a rate of 4 mL/h by microinfusion pump. Meanwhile, 20% mannitol (Tianjin Baxter Medical Supplies, Chinese drug standard H20003056) 250 mL, low molecular weight dextran (Shijiazhuang Four Pharmaceuticals, Chinese drug standard H13022484) 500 mL, and dexamethasone (Chinese Medicine Yung Sheng, Chinese drug standard H41020036) 10 mg were given intravenously.

### Statistical Analysis

Statistical analysis was performed using IBM SPSS version 22.0 (SPSS, Chicago, IL, USA). Data are expressed as the mean ± SD. The groups were compared using the independent sample *t*-test or the χ^2^ test as appropriate. Univariate analysis was performed to determine the association between angiographic or clinical results and other factors. Variables with *p* < 0.20 in univariate analysis were candidates for inclusion in multivariate logistic regression analysis. *p* < 0.05 was considered statistically significant.

## Results

Of the 5,126 patients who underwent cerebral angiography, 18 (0.35%) suffered transient cortical blindness. After the administration of medication, the vision of patients with transient cortical blindness was completely recovered within 2 h to 4 days.

### Risk Factors for Transient Cortical Blindness

As shown in Table 1, upon univariate statistical analysis, no significant differences were determined between the transient cortical blindness group and the control group regarding gender (*p* = 0.454), age (*p* = 0.872), and smoking (*p* = 0.170). The rates of hypertension were higher in the transient cortical blindness group than in the control group (55.6 vs. 36.8%), although this difference did not reach statistical significance (*p* = 0.100). Moreover, the transient cortical blindness group tended to have more patients with diabetes than the control group (11.1 vs. 6.8%), but again the difference between the two groups did not show statistical significance (*p* = 0.800). Compared with the control group, patients in the transient cortical blindness group weighed less (*p* = 0.020) and received a larger dose of contrast agent (*p* = 0.034), and more patients in this group had contrast agents injected into the posterior circulation (*p* < 0.001).

**Table 1:** Result from univariate statistical analysis for all variables.

**Variables**	**Control group (*n* = 5,108)**	TCB^*^ group (*n* = 18)	***P*-value**
Gender			0.454
Male	2,148	6	
Female	2,960	12	
Age	49.78 ± 11.56	50.22 ± 12.54	0.872
Weight	64.14 ± 11.16	60.00 ± 6.826	0.020
Smoking	1,514	8	0.170
Hypertension	1,881	10	0.100
Diabetes	348	2	0.800
Contrast agent dose	99.89 ± 32.07	115.94 ± 39.35	0.034
Contrast agent injected into			*P* <0.001
Anterior circulation only	2,061	3	
Posterior circulation and anterior circulation	2,896	11	
Posterior circulation only	151	4	

* *TCB, transient cortical blindness*.

### Multivariate Logistic Regression Analysis

Multivariate logistic regression analysis showed that dose of contrast agent and contrast agent injected into the posterior circulation were independent risk factors for transient cortical blindness (*p* < 0.05) (Table 2). Patients with larger contrast-agent dose tended to present with transient cortical blindness [odds ratio (OR) 1.011; 95% confidence interval (CI) 1.001–1.021; *p* = 0.036]. Compared with the contrast agent injected only into the anterior circulation, patients with contrast agent injected only into the posterior circulation had a significantly higher rate of transient cortical blindness (OR 18.976, 95% CI 4.188–85.977; *p* < 0.001).

**Table 2:** Multivariate logistic regression analysis.

**Variables**	**OR**	**95% CI**	***P*-value**
Contrast agent dose	1.011	1.001~1.021	0.036
Contrast agent injected into			<0.001
Anterior circulation only	Ref^*^		
Posterior circulation and anterior circulation	2.631	0.733~9.450	0.138
Posterior circulation only	18.976	4.188~85.977	<0.001

* *Ref, reference*.

## Discussion

Transient cortical blindness is characterized by loss of perceived vision, normal fundi, normal papillary reflexes, and unaltered extraocular movements (8, 9), and has been reported following cerebral angiography (10, 11). Symptoms may occur from the start of the cerebral angiography procedure until 12 h afterward (5). The recovery of normal vision may start within the first few hours, but complete recovery may take as long as 5 days (9, 12). In the present study, the vision of patients in the transient cortical blindness group was completely restored within 4 days. Although there have been many previous case reports or small case series of transient cortical blindness, few researchers have focused specifically on the risk factors for transient cortical blindness after cerebral angiography procedures (3–5). In this study we examined a much larger cohort who underwent cerebral angiography at five high-volume medical centers with the aim of identifying these risk factors. Upon univariate analysis, we found that the transient cortical blindness group weighed less and received a larger contrast-agent dose, with more patients having contrast agents injected into the posterior circulation. However, logistic regression analysis revealed that the dose of contrast agent and contrast agent injected into the posterior circulation were independent predictive factors for transient cortical blindness. This may be due to the characteristics of patient selection in our study or the dose–weight interactions that lead to contrast agents becoming more toxic with decreasing body mass.

The exact mechanism of transient cortical blindness remains unclear. The most popular hypothesis is contrast neurotoxicity. Some researchers have demonstrated that contrast agents may induce vasodilation, shrinkage of cerebrovascular endothelial cells, and widened endothelial tight junctions (13–15). Moreover, previous studies found that the risk of transient cortical blindness increases when the contrast agent in the cerebral circulation is higher (9, 16). A higher dose of contrast agent could prolong the duration of exposure of the cerebrovascular endothelium to the contrast agent by increasing the transit time, thus resulting in blood–brain barrier breakdown, which would further increase the transfer of contrast material (17). After the contrast agent penetrates the cerebral cortex, it could lead to adverse effects on the neurons and transient cortical blindness. The results of the present study, whereby we identified a larger contrast-agent dose as an independent predictive factor, confirm these findings.

Chronic hypertension may be also a factor that contributes to the breakdown of the blood–brain barrier and transient cortical blindness. Chronic hypertension could impair the cerebral arterioles and subsequently lead to hypoperfusion, which would then result in brain ischemia and subsequent vasogenic edema (18). The results of the current study further confirm the correlation between hypertension and transient cortical blindness. The rates of hypertension were higher in the transient cortical blindness group than in the control group (55.6 vs. 36.8%), although this difference did not reach statistical significance (*p* = 0.100).

Previous studies reported that injection of contrast agent into posterior cerebral arteries plays an important role in the development of transient cortical blindness. The largest series of cortical blindness after cerebral angiography reported an incidence of 0.3–1%, with the highest incidence being reported following vertebral angiography (12). Tong et al. reviewed 12 cases with transient cortical blindness after endovascular procedures (19). In seven patients, an endovascular procedure was performed only in the vertebrobasilar arterial system whereas in five patients, the procedure was performed in both the carotid arteries and the vertebrobasilar arterial system. Possible reasons offered were that the posterior cerebral circulation had less extensive sympathetic innervation than the anterior circulation, and the lack of protective arterial vasoconstriction could thus act as a trigger for the breakdown of the blood–brain barrier when the contrast medium was administered (20, 21). Our study also confirmed that patients with contrast agents injected into the posterior circulation in the transient cortical blindness group numbered significantly more than in the control group (*p* < 0.001).

Other factors, such as diabetes, may make patients susceptible to the risk of transient cortical blindness. Recurrent hypoglycemia in diabetic patients could result in brain ischemia and brain edema caused by the diminished glucose levels and insufficient energy supplies (22). Hypoglycemia could also increase intracellular electrolytes, causing water retention and edema, which cause neuronal damage (23). Furthermore, Reivich et al. reported that the glucose consumption of the occipital cortex was the highest in the human body, which may cause further damage to the visual cortex in hypoglycemia (24). These pathophysiologic changes may be the predisposing causes of transient cortical blindness. Similarly, in our study, the rates of diabetes in patients were higher in the transient cortical blindness group than in the control group (11.1 vs. 6.8%), although the difference was not statistically significant (*p* = 0.800). This might be attributed to the small sample size in our study.

Autoimmune diseases may be also a predisposing factor for developing transient cortical blindness. Vasospasm and vasogenic edema in the parietal/occipital regions have been identified in patients with autoimmune diseases, increasing patients' susceptibility to neurotoxic effects of contrast media, thus resulting in transient cortical blindness (25). However, in our study none of the patients with transient cortical blindness suffered from autoimmune diseases. This might be due to the limited sample size and patient selection bias, as only patients with cerebral angiography were included.

No particular management strategy has yet been proved effective for improving the natural history of transient cortical blindness following cerebral angiography (9, 26) However, physicians should be aware that transient cortical blindness is self-limiting and reversible. When it does occur, patients should be reassured of the excellent prognosis of this condition and that symptoms will resolve spontaneously within a few days. In our study, according to our experience, after cerebral vessel dilation, cerebral edema alleviation, volume expansion, and steroid administration, the vision of patients in the transient cortical blindness group was completely restored within 4 days. Moreover, according to the results of our study, the risk of transient cortical blindness may be decreased by reducing the dose of contrast agent and paying more attention to the patients of lighter weight, representing future useful reference points for physicians.

There are some limitations to our study. First, owing to its retrospective nature and limited number of patients with transient cortical blindness, the data may be insufficient for generalization. Moreover, a limited number of factors were analyzed. More data on larger numbers of patients with transient cortical blindness and a wider variety of factors are required. Lastly, we did not take encephalopathy into consideration because contrast-induced encephalopathy could manifest as cortical blindness.

## Conclusion

This multicenter cohort study identified a larger dose of contrast agent and contrast agent injected into the posterior circulation as independent predictive factors for transient cortical blindness. These findings need to be confirmed by more prospective, large, multicenter, multipopulation studies.

## Data Availability Statement

All datasets generated for this study are included in the manuscript/supplementary files.

## Ethics Statement

The studies involving human participants were reviewed and approved by the ethics committee of the China-Japan Union Hospital of Jilin University. The patients/participants provided their written informed consent to participate in this study.

## Author Contributions

ML contributed to the preparation of the manuscript and data collection. HLia, CL, HLiu, YZ, and WS contributed to data analysis and interpretation. JW contributed to the experimental design and manuscript revision.

### Conflict of Interest

The authors declare that the research was conducted in the absence of any commercial or financial relationships that could be construed as a potential conflict of interest.
